# New nurse workplace adaptation: a Walker and Avant concept analysis

**DOI:** 10.3389/fpubh.2025.1713343

**Published:** 2025-12-10

**Authors:** Longhui Xu, Xiao Cong, Jinfang Wang, Renxiu Wang, Mei Yu, Lijing Zhang, Yuanjie Liu, Cuiping Xu

**Affiliations:** 1 Department of Nursing, The First Affiliated Hospital of Shandong First Medical University & Shandong Provincial Qianfoshan Hospital, Jinan, China; 2 School of Nursing, Shandong University of Traditional Chinese Medicine, Jinan, China; 3 School of Nursing, Shandong First Medical University & Shandong Provincial Academy of Medical Sciences, Jinan, China; 4 Hospital Vice President’s Office, The First Affiliated Hospital of Shandong First Medical University & Shandong Provincial Qianfoshan Hospital, Jinan, China

**Keywords:** adaptation, burnout, nurses, personnel turnover, professional role, socialization, workplace

## Abstract

**Introduction:**

High turnover among new nurses critically threatens the stability of the global healthcare workforce. While existing theories explain why new nurses struggle, they fail to clarify how new nurses successfully adapt. This gap shifts the focus to the individual-driven process of workplace adaptation, a key factor in retention. However, the core concept of workplace adaptation itself remains ambiguous and is often conflated with top-down organizational socialization. Without a clear and consistent definition, progress in understanding and facilitating this critical process is severely limited. Accordingly, this study aims to systematically clarify the concept of new nurse workplace adaptation.

**Methods:**

Concept analysis was conducted using Walker and Avant’s eight-step method. A systematic search of CNKI, Wanfang Data, CQVIP, CBM, CiNii, PubMed, Web of Science, CINAHL, Embase, and PsycINFO was conducted from their inception to June 1, 2025.

**Results:**

The attributes of new nurse workplace adaptation were identified as continuous dynamic adjustment, achievement of internal–external equilibrium, and integration into team collaboration. Antecedents include sociodemographic factors, individual intrinsic adaptation capital, and organizational socialization strategies. Consequences encompass promotion of personal career development, optimization of nursing service quality, and stabilization of team organizational structure.

**Discussion:**

This concept analysis clarifies new nurse workplace adaptation as an integrated, individual-driven process, distinct from top-down organizational socialization. This distinction is critical as it reveals the overlooked phenomenon of “sacrificial adaptation”—a state where a nurse compromises internal well-being for superficial integration. The identification of this phenomenon exposes a fundamental flaw in management strategies that prioritize behavioral compliance over authentic well-being. This ultimately points to the necessity of a paradigm shift: from enforcing conformity to cultivating supportive environments where genuine adaptation can thrive.

## 1 Introduction

The global nursing shortage presents a significant structural challenge to healthcare systems. The World Health Organization estimates a global shortfall of 4.5 million nurses and 0.31 million midwives by 2030 (1). More alarming than this future deficit is the current erosion of stability within the active nursing workforce: the global nurse turnover rate is approximately 15.2%, with the most critical regions for attrition being the Eastern Mediterranean (23.1, 95% *CI*: 7.7–52.0%), followed by the Western Pacific (17.8, 95% *CI*: 9.8–30.3%), the Americas (13.4, 95% *CI*: 10.7–16.8%), and Europe (6.3, 95% *CI*: 1.9–18.6%) (2). Against this backdrop, the retention of new nurses—a crucial strategic reserve—critically impacts the stability of the entire nursing human resource system (3). However, a staggering 31.9% of new nurses leave within their first year, and another 25.0% within the second (4). This indicates that the new nurse cohort, intended as a “strategic buffer,” has become a primary source of workforce instability. Their frequent departures not only force healthcare institutions into a vicious cycle of “recruitment-training-departure-re-recruitment,” rendering substantial upfront human capital investments as sunk costs (4), but also exacerbate the workload and burnout of experienced nurses, ultimately damaging organizational efficiency and competitiveness (5). Therefore, addressing new nurse retention is not merely an organizational task but a strategic cornerstone for ensuring healthcare system integrity and public health security.

New nurses are in a transition period from student to professional, often experiencing confusion and self-doubt, which increases their tendency to leave (6, 7). The reasons for this are not attributed to individual capability but rather to a systemic dilemma caused by the theory-practice gap. As early as the 1970s, Kramer (8) defined this phenomenon as “reality shock” to explain and intervene in high new nurse turnover, advocating a shift from blaming “individual failure” to solving “systemic disconnection.” Building on this, Duchscher (9) proposed the “transition shock” model, a comprehensive framework incorporating the factors and experiences of role transition. However, both “reality shock” and “transition shock” theories predominantly offer diagnostic explanations for “why new nurses are trapped” during their transition period, failing to fully address the critical practical question of “how to escape.” Consequently, a profound explanation of the differentiation in new nurses’ career trajectories requires shifting the focus of research from the common impact of external shocks to the differential process by which individuals actively mobilize resources to cope with internal and external challenges. This dynamic adjustment, powered by individual agency, is precisely the core phenomenon captured by the concept of “new nurse workplace adaptation” (10–12). Existing research indicates that successful workplace adaptation can effectively alleviate transition-related stress (13) and significantly reduces new nurses’ turnover intention (12), further solidifying the status of new nurse workplace adaptation as a core variable influencing the stability of the nursing workforce.

Currently, the concept of new nurse workplace adaptation is unclearly defined in academic literature, with its ontological status subject to diverse interpretations—as a process (14–16), a state (17–19), or a capability (20–22)—and its boundaries often blurred with related concepts like organizational socialization. This conceptual ambiguity leads to a lack of consensus among researchers when selecting measurement methods and defining success criteria, making it difficult to effectively integrate and compare findings from different studies, and weakening the possibility of extracting generalizable experiences and guidance from existing literature. Therefore, clarifying and systematically defining the concept of new nurse workplace adaptation has become an urgent priority for advancing in-depth research in this field. Concept analysis is a methodology that can systematically clarify concepts, promote theoretical development, and enhance the rigor of research and the effectiveness of practical interventions (23). Given the well-established and efficacious nature of Walker and Avant’s method (24) for systematically analyzing such concepts, this study adopts this methodology. Through steps such as identifying core attributes and constructing cases, this study aims to systematically clarify the intension and extension of new nurse workplace adaptation, while highlighting the unique context and role demands of the new nurse cohort, in order to lay a solid theoretical foundation for subsequent empirical research.

## 2 Methods

### 2.1 Concept analysis method

This study employed Walker and Avant’s method (24) to analyze the concept of new nurse workplace adaptation. The method comprises eight distinct steps: (1) identifying the concept; (2) clarifying the purpose of the concept analysis; (3) determining the concept’s application in the literature; (4) identifying the defining attributes; (5) constructing a model case; (6) constructing borderline and contrary cases; (7) analyzing antecedents and consequences; and (8) providing empirical referents.

### 2.2 Data sources

To comprehensively gather relevant research, we systematically searched Chinese literature databases (CNKI, Wanfang Data, CQVIP, CBM) and English literature databases (PubMed, Web of Science, CINAHL, Embase, PsycINFO). We also included the Japanese literature database CiNii to incorporate East Asian perspectives comparable to the Chinese context, while other non-English sources (e.g., Korean, Spanish) were excluded due to language barriers. Our search strategy combined subject headings and free-text keywords, with field limitations applied, and the time frame set from the database inception to June 1, 2025. The core keywords primarily revolved around two sets of concepts (adapted to the respective database languages): first, terms referring to new nurses, such as “new nurse*,” “newly graduated nurse*,” “newly qualified nurse*,” “newly employed nurse*,” “newly registered nurse*,” “newly licensed nurse*,” “newly trained nurse*,” “neophyte nurse*,” “junior nurse*,” and “novice nurse*”; and second, vocabulary describing workplace adaptation concepts, such as “workplace adaptation,” “workplace adaptability,” and “workplace adjustment.” Additionally, we utilized a snowballing technique to trace references from key articles and consulted dictionaries, thesauri, and textbooks to ensure the comprehensiveness of our retrieval.

### 2.3 Data collection

**Figure 1:**
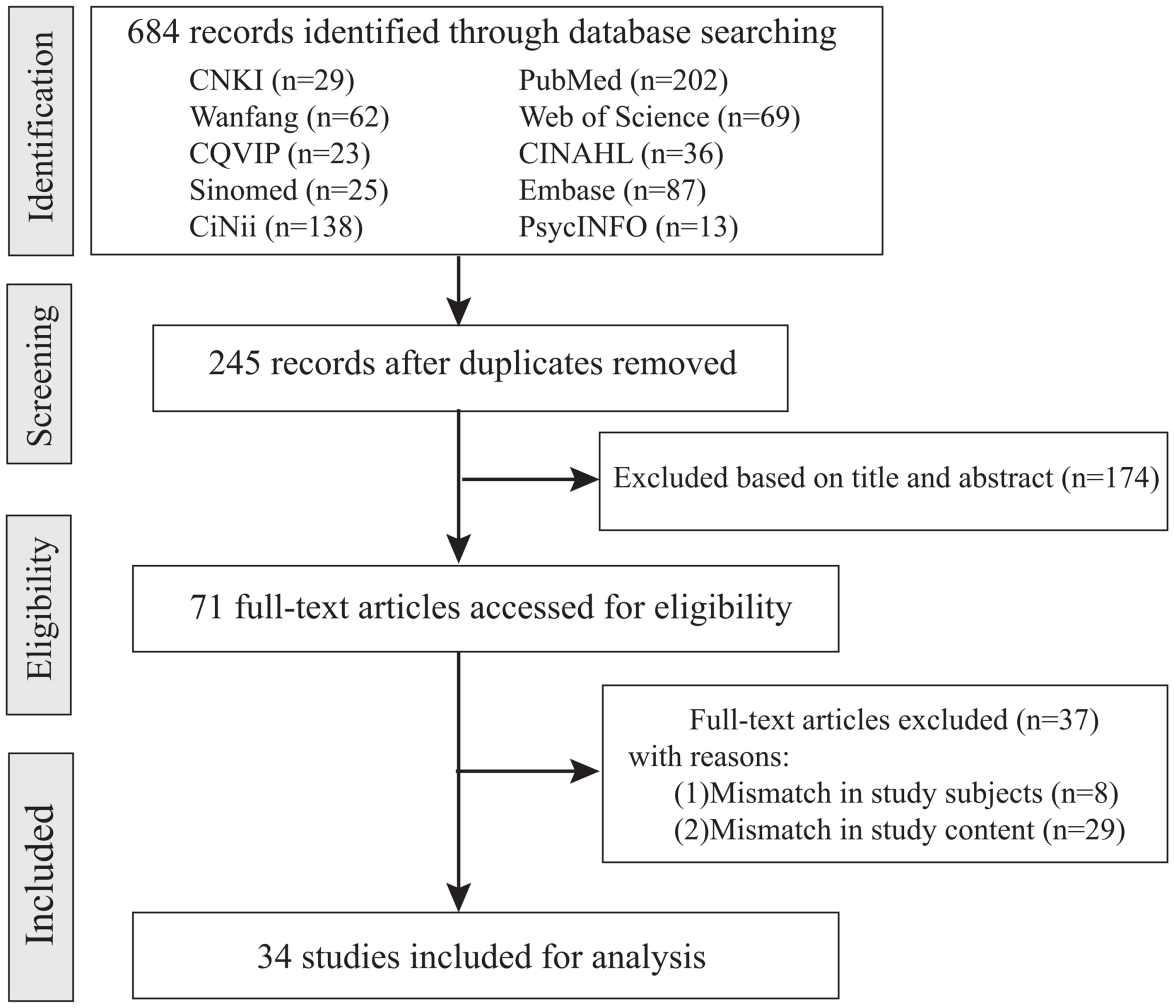
Flowchart of the study selection process of the concept analysis.

The inclusion criteria for the literature were: (1) content pertaining to the conceptual definition, defining attributes, concept clarification, antecedents, consequences, and measurement tools related to new nurse workplace adaptation; and (2) publication in Chinese, Japanese, or English. The exclusion criteria were: (1) duplicate publications; (2) incomplete or inaccessible full-text articles; and (3) conference papers, editorials, and letters to the editor. Two researchers independently screened the literature, and any discrepancies were resolved through discussion with a third researcher. An initial search across ten databases identified a total of 684 relevant articles. After removing duplicates, 245 articles remained. Following the screening of titles and abstracts, 174 articles were excluded. A further 37 articles were excluded after full-text review, resulting in a final selection of 34 articles for concept analysis. The literature screening process is illustrated in Figure 1, and detailed information on the included literature is provided in Supplementary Table S1.

## 3 Results

### 3.1 Uses of the concept

#### 3.1.1 Dictionary definition

“Workplace adaptation” is a phrasal noun comprising two sub-concepts: “workplace” and “adaptation,” both of which require individual identification. Definitions of “workplace” typically begin with its physical attributes. Dictionaries like Cambridge (25) and Merriam-Webster (26) describe it as a tangible location—such as an office, factory, or shop—where people work. A broader perspective is offered by the Oxford English Dictionary (27), which defines it as “the site or location where a person works…associated with a company or enterprise,” highlighting the close functional or organizational ties between these work settings and specific companies. This focus on a professional context is also apparent in definitions from other languages: the Modern Chinese Dictionary (28) terms it a place for work or taking up a post, and the Weblio Japanese Dictionary (29) calls it “a place for professional work.” Collectively, the core meaning of “workplace” points to the physical space where an individual engages in occupational activities, with its extension often encompassing the organizational context, functional attributes, and the social and economic activities it supports.

Regarding “adaptation,” English dictionaries (25–27) consistently define it as the process of changing to suit new or different conditions, emphasizing its dynamic response to the environment. This concept is further nuanced by other perspectives: the Modern Chinese Dictionary (28) focuses on the outcome, defining adaptation as the achievement of congruence by “suit[ing] (objective conditions or needs)”; the Weblio Japanese Dictionary (29) provides a psychological and sociological interpretation, describing it as a change in “behavior or consciousness”; and the APA Dictionary of Psychology (30) elucidates the underlying cognitive mechanisms, such as the processes of “assimilation and accommodation.” In summary, “adaptation” is a complex construct referring to the process by which an individual or system adjusts and changes in structure, function, behavior, or cognition to cope with environmental changes and meet specific needs, with the goal of achieving a new equilibrium with the environment (including physical, social, and psychological aspects).

#### 3.1.2 Definitions of terms in literature

Scholarly definitions of new nurse workplace adaptation can be categorized into four main perspectives:

(1) *A dynamic process*. This viewpoint frames adaptation as a time-bound process of integration and identity formation. It is described as the period during which new nurses acquire the necessary roles and attitudes of their profession (14), establish a professional identity through environmental interaction (15), and achieve psychological integration by internalizing professional values (16).
(2) *A state of equilibrium*. A second perspective defines adaptation as a state of equilibrium between the individual and the environment. This balance is achieved either through continuous adjustment to maintain physical and mental well-being (17), via active “cognitive restructuring” based on Piaget’s theory (18), or is experienced as a subjective psychological state of harmony with the workplace (19).
(3) *A cultivatable capability*. The third viewpoint, prominent in Chinese literature, conceptualizes adaptation as an ability to adapt to workplace norms and build relationships (20), which has been expanded to encompass core professional competencies and the self-regulation skills needed to solve complex problems (21, 22).
(4) *A multidimensional construct*. The fourth perspective views adaptation as a multidimensional construct, with scholars identifying dimensions that range from broad categories like professional identity, lifestyle, and technical skills (31) to more granular components such as interpersonal relationships, work tasks, and environmental factors (32).

### 3.2 Determining the defining attributes of the concept

Defining attributes are the critical characteristics of a concept, representing recurring conceptual features, elements, or component characteristics found in the literature (24).

#### 3.2.1 Continuous dynamic adjustment

New nurse workplace adaptation is not a fixed endpoint state or an isolated event, but rather a continuous and dynamic process of adjustment (17, 32). New nurses entering clinical practice frequently encounter a variety of complex challenges, experiencing pressure and impact on multiple levels, including physiological and psychological. They must continuously engage in cognitive restructuring, emotional regulation, and behavioral learning, while gradually internalizing the professional norms, behavioral expectations, and work culture of their healthcare organization, to successfully transition from student to practicing nurse (19).

#### 3.2.2 Achievement of internal–external equilibrium

Upon entering the workplace, new nurses often experience conflicts or imbalances due to mismatches between their internal systems (e.g., existing expectations, abilities, values) and the external work environment systems (e.g., workload, leadership style, organizational norms) (33, 34). “Achievement of internal–external equilibrium” refers to the state where new nurses, by integrating new work experiences and demands into their existing cognitive-behavioral frameworks (assimilation) and adjusting and modifying their original cognitive-behavioral frameworks based on new experiences and demands (accommodation), ultimately achieve a stable state of harmonious and efficient operation between their internal cognition, abilities, behavior, and the requirements of the external work environment (18).

#### 3.2.3 Integration into team collaboration

“Integration into team collaboration” is manifested on two levels. Firstly, on a psychosocial level, new nurses clearly understand their roles, responsibilities, and value within the team, establish positive emotional connections with team members, and adapt to the team’s unique subculture and informal norms (18, 32). Secondly, on a practical work level, new nurses effectively collaborate with team members, engaging in efficient communication, resource coordination, and mutual support to achieve common goals (18, 32).

### 3.3 Identifying cases

To concretely illustrate the defining attributes and clearly delineate the conceptual boundaries of new nurse workplace adaptation, we constructed a model case, a borderline case, and a contrary case (24). These cases were synthesized based on observations conducted by the research team under the guidance of a hospital vice president and several head nurses with extensive management experience in tertiary public hospitals in China. The settings include both tertiary public hospitals and community health centers, incorporating labor market factors such as involuntary employment choices. The characters in the cases represent the typical demographic profile of new nurses in China: aged 22–24, holding a bachelor’s degree, and employed in their current position for ≤1 year with no prior full-time experience in other hospitals.

#### 3.3.1 Model case

Newly graduated nurse Xiao Zhong commenced her role at a tertiary hospital, immediately confronting the rigorous challenges of high-intensity work and complex clinical situations. She had previously received stern criticism from hospital leadership due to a procedural error. Despite this setback, she did not become discouraged. Instead, she dedicated her off-duty hours to diligently studying professional knowledge, humbly sought advice from senior colleagues, and repeatedly honed her nursing skills. As she grew more familiar with the department’s environment, she began observing and emulating her colleagues’ specific routines and habits in executing medical orders, during shift handovers, and in inter-professional communication, striving to move from rote application to flexible utilization (continuous dynamic adjustment). After several months of dedicated effort, Xiao Zhong could calmly manage daily tasks and respond to emergencies. The fear and self-doubt she initially harbored about the high-pressure environment began to dissipate, replaced by a grounded sense of quiet confidence and self-assurance (achievement of internal–external equilibrium). Concurrently, she actively integrated into the department’s daily interactions and mutual support, engaging in effective communication and seamless coordination with team members during every rescue operation. These shared experiences of working together solidified her sense of belonging within this busy yet supportive collective (integration into team collaboration).

#### 3.3.2 Borderline case

Newly graduated nurse Xiao Luo joined the cardiovascular surgery department of a general hospital, a field renowned for its high specialization and rapid pace. He utilized his off-duty hours to read specialized books and actively participated in various in-hospital skill training sessions. He quickly mastered common nursing procedures and instrument usage in cardiovascular surgery, rapidly adapting to the department’s shift schedules and workload. Facing daily work and emergent situations, Xiao Luo grew increasingly composed, and his work confidence also improved (good demonstration of continuous dynamic adjustment). However, despite his flawless task performance, Xiao Luo felt a constant sense of alienation. To avoid conflict and meet organizational expectations, he chose to suppress his true feelings and mechanically follow the team’s social scripts. While he appeared compliant on the surface, he internally struggled with a sense of inauthenticity and exhaustion, feeling like an actor wearing a mask rather than a genuine team member (partial achievement of internal–external equilibrium). He strictly limited his interactions to task-related communication and minimal social necessity, and meticulously maintained professional boundaries. This transactional approach prevented the formation of deep emotional connections or team synergy, resulting in him gaining little sense of belonging or recognition derived from true team integration (significant deficiency in integration into team collaboration).

#### 3.3.3 Contrary case

Xiao Zhou joined a community health center after failing her postgraduate entrance examination, a role she perceived as unchallenging and limiting. For 3 months, she lacked enthusiasm for learning, frequently complained about the tedious work and low salary, and showed no initiative to improve her skills (lack of continuous dynamic adjustment). Her accumulated resentment led to a perfunctory attitude towards regulations and tasks, deepening her sense of unfulfilled potential and preventing any form of internal balance (failure to achieve internal–external equilibrium). Socially, she remained on the periphery of the team, showing indifference to colleagues and believing herself to be out of place (failure to integrate into team collaboration). Ultimately, Xiao Zhou voluntarily resigned.

### 3.4 Antecedents

Antecedents refer to the preceding factors or events that influence the workplace adaptation of new nurses (24).

#### 3.4.1 Sociodemographic factors

Sociodemographic factors are the inherent personal attributes that new nurses bring to the workplace, forming the initial conditions for their adaptation. These include:

(1) *Gender*. From a group characteristics perspective, male nurses may exhibit certain advantages in physically demanding tasks, which could serve as a positive factor for adapting to high-workload clinical practice (35, 36).
(2) *Educational background*. Nurses with higher academic qualifications often have stronger learning abilities, facilitating their adaptation (37). However, this background may also foster elevated professional expectations that, especially when combined with labor market constraints (e.g., involuntary placement), can intensify the misalignment with workplace realities and create adaptation challenges (38).
(3) *Upbringing*. Growing up in diverse environments may equip new nurses with more flexible cognitive patterns and a greater openness to experience, enhancing their adaptability to the nursing work environment (39).

#### 3.4.2 Individual intrinsic adaptation capital

A new nurse’s intrinsic adaptation capital consists of the personal resources accumulated prior to employment that are crucial for navigating transitional challenges. These include:

(1) *Professional competence*. Solid clinical skills and knowledge allow nurses to build professional confidence by completing tasks safely and efficiently, thereby earning trust from patients and colleagues and accelerating team integration (14, 40).
(2) *Interpersonal skills*. Proficient interpersonal skills help new nurses navigate the shift from academic to professional relationships by enabling them to quickly discern workplace rules, reduce communication-related stress, and establish collaborative ties more smoothly (14, 19, 35).
(3) *Psychological capital*. Positive psychological states developed over time, such as resilience and optimism, enable new nurses to maintain a positive outlook during adversity, proactively seek solutions, and effectively overcome work challenges (12, 41, 42).

#### 3.4.3 Organizational socialization strategies

Organizational socialization strategies are the institutional supports designed to facilitate new nurses’ integration. Key strategies include:

(1) *Mentorship*. Senior colleagues transfer valuable, tacit clinical experience through instruction and demonstration, effectively bridging the critical theory-practice gap for novices (15, 18).
(2) *Humanistic care*. Supportive leadership from nurse managers fosters psychological safety, which empowers new nurses to explore, ask questions, and seek help in high-pressure environments, thus accelerating their role transition (39).
(3) *Work atmosphere*. A supportive or magnetic work environment promotes adaptation by fostering professional autonomy, offering rich developmental opportunities, and enhancing a sense of workplace belonging (12, 43, 44).
(4) *Workload management*. Scientific management of schedules, working hours, and compensation helps mitigate occupational fatigue and fosters a perception of balanced effort and reward, which is fundamental to successful adaptation (11, 35, 45).

### 3.5 Consequences

Consequences refer to the potential events or situations that may arise following the workplace adaptation of new nurses (24).

#### 3.5.1 Promotion of personal career development

Well-adapted new nurses are better equipped to manage occupational stressors (such as anxiety and frustration), which facilitates a smoother transition from student to practitioner (21, 22). They tend to formulate more realistic career plans and more readily identify opportunities for professional growth within the organization, a process that contributes to greater self-efficacy and professional gain, ultimately exhibiting lower levels of burnout and turnover intention (18, 21, 46, 47).

#### 3.5.2 Optimization of nursing service quality

New nurses who adapt successfully demonstrate a stronger commitment to their professional role, which translates into more dedicated engagement in clinical work (48). This dedication is evident in their practice, where they may actively use narrative methods and deep listening to better understand patients (48). Moreover, successful workplace adaptation contributes to enhancing new nurses’ clinical leadership. Increased leadership, in turn, positively influences patient-provider communication, nurse–nurse trust, and nurse-physician collaboration, reinforcing their workplace adaptation. This ultimately creates a self-reinforcing virtuous cycle that drives the development of high-quality nursing services (13).

#### 3.5.3 Stabilization of team organizational structure

Well-adapted new nurses quickly internalize team collaboration norms and communication patterns, establishing efficient working relationships that reduce internal conflict and communication costs (18, 46). Crucially, successfully adapted nurses also have a lower intention to leave. This higher retention rate helps break the vicious cycle of “recruitment-training-departure,” alleviating the burnout experienced by senior nurses from continuous mentoring. This stability provides a fundamental safeguard for the intergenerational transmission of valuable clinical experience and the long-term health of the entire nursing team (12, 40).

### 3.6 Measurement tools for new nurse workplace adaptation

Empirical referents are used to measure the manner or degree to which a concept exists (24). Currently, two scales are used to assess new nurse workplace adaptation.

#### 3.6.1 Nurses’ workplace adaptability scale (NWAS)

The NWAS, developed by Fujimoto and Shizuko (49), is designed to assess nurses’ workplace adaptability. This 20-item scale is rated on a 5-point Likert format and comprises four 5-item dimensions: business autonomy, relationship with supervisor, workplace atmosphere, and environmental integration. This scale demonstrated good internal consistency, with a total Cronbach’s *α* of 0.86 and Cronbach’s α values for the dimensions ranging from 0.69 to 0.84.

A Chinese version was later adapted by Liu et al. (20). This 17-item adaptation, also using a 5-point scale, features three dimensions: work environment and atmosphere (11 items), relationship with supervisor (4 items), and business autonomy (2 items). This version was validated on a sample of new nurses and demonstrated good internal consistency (total Cronbach’s *α* = 0.89; dimensions Cronbach’s *α* ranged from 0.60 to 0.90), confirming its suitability for this specific population.

#### 3.6.2 Workplace adaptation behavior and state scales for new nurses (WABSS-NN)

The WABSS-NN, developed by Kitajima and Hosoda (18), is uniquely grounded in Piaget’s theory and provides a comprehensive assessment by using two distinct sub-scales, both rated on a 5-point Likert scale.

(1) The workplace adaptation behavior sub-scale is a 34-item measure of adaptive actions. It includes five dimensions: utilization of human resources (10 items), proactive work engagement (8 items), effort to become part of the team (7 items), self-study of necessary professional knowledge (5 items), and understanding of job roles (4 items). Its internal consistency is high (total Cronbach’s *α* = 0.93; dimensions Cronbach’s *α* ranged from 0.76 to 0.91).
(2) The workplace adaptation state sub-scale is a 26-item measure of perceptual and psychological outcomes. It assesses four dimensions: sense of efficacy in nursing practice (10 items), positive work outlook (7 items), sense of belonging to the team (6 items), and awareness of independence (3 items). It also demonstrated excellent internal consistency (total Cronbach’s *α* = 0.95; dimensions Cronbach’s *α* ranged from 0.82 to 0.93).

A comparison of these instruments with our proposed attributes reveals key differences regarding conceptual overlap and depth. The WABSS-NN’s Behavior sub-scale sufficiently captures the “continuous dynamic adjustment” and partly addresses the “integration into team collaboration” attributes. However, both scales show limitations in fully measuring the “internal–external equilibrium.” In operationalizing equilibrium, the existing scales predominantly focus on either observable coping behaviors or resultant emotional outcomes (e.g., sense of belonging). They lack specific items designed to assess the depth and stability of this internal harmonization, particularly the extent to which the nurse has achieved authentic equilibrium versus sacrificial compliance. Therefore, future tool development must target the psychological negotiation of values and expectations.

### 3.7 Definition of the concept

**Figure 2:**
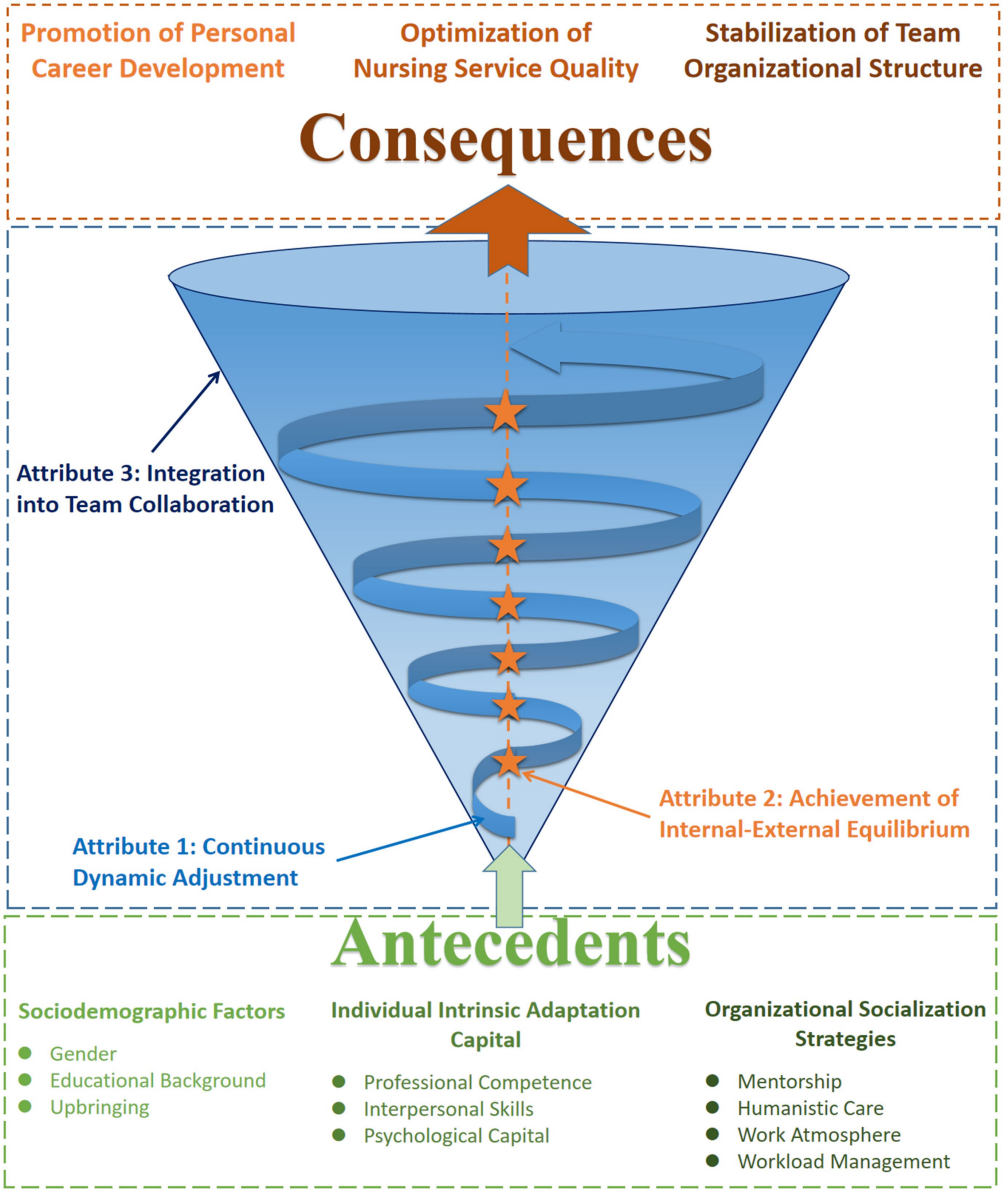
The integrated conceptual model of new nurse workplace adaptation. The blue spiral represents continuous dynamic adjustment; the orange stars denote internal–external equilibrium; and the funnel background depicts integration into team collaboration. The trajectory rises from antecedents (bottom) to consequences (top).

New nurse workplace adaptation is defined as the continuous dynamic adjustment process undertaken by nursing personnel in their initial positions after licensure to facilitate role transition. This process is characterized by the harmonization of internal cognitive-behavioral frameworks with external work environment systems, and the effective integration of team role norms and member relationships, ultimately achieving a synergistic alignment between personal development and organizational goals. An integrated conceptual model of new nurse workplace adaptation is shown in Figure 2.

## 4 Discussion

Unlike previous research that has viewed workplace adaptation as an isolated process (14–16), a state (17–19), or a capability (20–22), this study posits that it is an indivisible, complex construct. Its core lies in the organic unity of three defining attributes: continuous dynamic adjustment, achievement of internal–external equilibrium, and integration into team collaboration. Specifically, (1) continuous dynamic adjustment is the foundational process of learning and behavioral modification that new nurses undertake to achieve role transition; (2) achievement of internal–external equilibrium is the goal state, signifying a harmonious state where a new nurse’s internal cognition, emotions, and behaviors are matched with the demands of the external work environment; and (3) integration into team collaboration is the indispensable social dimension, reflecting an individual’s ability and performance in establishing positive relationships, sharing resources, and jointly achieving goals within team interactions. These attributes are closely interconnected and mutually supportive: antecedents (sociodemographic factors, individual intrinsic adaptation capital, and organizational socialization strategies) drive new nurses to engage in continuous dynamic adjustment. Successful adjustment, in turn, leads to a state of internal–external equilibrium and facilitates the smooth integration of new nurses into the team. Subsequently, effective team integration can further optimize adjustment strategies, reinforcing the state of equilibrium and culminating in a virtuous cycle that is validated by positive consequences (promotion of personal career development, optimization of nursing service quality, and stabilization of team organizational structure).

It is crucial to acknowledge the potential trade-offs when exploring how new nurses achieve “internal–external equilibrium” and “integration into team collaboration.” As seen in the borderline case of Xiao Luo, his strategy of “low-profile survival”—suppressing his self and sacrificing some interpersonal interactions—allowed him to achieve a degree of work-skill equilibrium but failed to bridge the gap with his team. This phenomenon of “sacrificial adaptation” is not uncommon. Research shows that new nurses, to survive in challenging hierarchical environments, may make compromises like suppressing emotions or flexibly modifying their methods (50). Some adopt a “chameleon” role to integrate, suppressing their true selves and even rationalizing inappropriate behaviors (51). The underlying reason may be a conflict between an individual’s self-esteem needs, which drive the subjective pursuit of internal equilibrium, and the need for love and belonging, which relies on objective team evaluation (52). When the perception of subjective equilibrium diverges from the team’s standard of objective integration, new nurses may experience cognitive dissonance and engage in regressive adaptation to prioritize the satisfaction of love and belonging needs. Such adaptation strategies, achieved at the cost of self and principles, are not a path to healthy professional growth but rather passive compromises made in less-than-ideal environments, contradicting our expectation for new nurses to actively integrate and contribute professionally. Therefore, in research and practice, we must not only focus on how new nurses adapt but also reflect on and improve the environmental factors that compel them to sacrifice their true selves.

This study views the process of new nurse integration into an organization as, essentially, a complex reciprocal interaction between the organization and the individual. To precisely deconstruct this interaction, it is first necessary to distinguish between two complementary concepts: organizational socialization and workplace adaptation. Organizational socialization is a top-down, organization-driven process that uses strategies to shape “outsiders” into compliant “insiders” (53, 54). As highlighted by organizational psychology frameworks, such as Ashforth and Saks’s typology (55), this process is characterized by institutional tactics deployed to suppress the individuality of newcomers and facilitate their adoption of predefined organizational norms and behavioral compliance. In contrast, workplace adaptation is a bottom-up, individual-driven process where new nurses use cognitive and psychological integration to achieve a dynamic balance between the individual and the environment. For a long time, management has tended to focus on the organizational perspective, viewing new nurses’ compliance with norms as a marker of successful integration. However, behavioral socialization cannot conceal potential psychological adaptation failures in new nurses (56, 57). When external behaviors become disconnected from internal emotions, the strong sense of organizational injustice experienced by new nurses can induce workplace deviance—that is, resisting the organization and seeking psychological rebalancing through methods such as passive resistance and spreading negative remarks (58). Such behaviors not only erode organizational efficiency and stability but also severely damage the psychological well-being and occupational safety of other nurses, ultimately jeopardizing patient welfare and the normal functioning of the entire healthcare system (59). Therefore, to fundamentally resolve the difficulties in new nurse integration and retention challenges, managers urgently need to undergo a profound “cognitive transformation,” shifting the management focus from unilateral organizational socialization and regulation to a deep concern for and support of individual workplace adaptation quality (60). This implies that organizations should strive to build a more inclusive and supportive environment, establishing open communication channels and effective feedback mechanisms to listen to the genuine voices of new nurses, help them successfully navigate their transition period, and achieve mutual success for individuals and the organization.

## 5 Limitations

While this conceptual analysis provides a foundation for this paradigm shift, its limitations highlight crucial directions for future inquiry. First, although this study incorporated multinational literature, linguistic constraints may have precluded a fully universal understanding of the concept. Future research should therefore broaden its scope to better define the commonalities and specificities of new nurse adaptation across cultures. Second, the interpretation of the concept’s attributes was inevitably shaped by the cultural contexts of the analyzed literature. Specifically, the strong emphasis on “integration into team collaboration” may reflect collectivist norms prevalent in East Asian healthcare settings. In contrast, individualistic cultures often prioritize personal autonomy and task performance over team synergy, which may alter the concept’s practical manifestation. Consequently, we strongly recommend future comparative, cross-cultural studies to test the validity and applicability of the proposed model in diverse settings. Such work is essential for deepening the cross-cultural understanding of new nurse workplace adaptation and ultimately achieving the goal of mutual success for both individuals and organizations.

## 6 Conclusion

This concept analysis contributes a more integrated definition of new nurse workplace adaptation, moving beyond isolated views of it as a process, a state, or a capability. Crucially, this reframing distinguishes individual-driven adaptation from top-down organizational socialization, bringing to light the critical yet overlooked phenomenon of “sacrificial adaptation”—where new nurses compromise personal well-being for superficial integration. The primary implication is a call for a paradigm shift in management: from enforcing compliance to genuinely fostering individual adaptation by creating supportive work environments. Future research, guided by frameworks like the Job Demands-Resources model, should prioritize cross-cultural, longitudinal studies to develop strategies that promote healthy, sustainable adaptation, ultimately securing both the well-being of new nurses and the stability of the global healthcare workforce.
