# Supplementary material for: New nurse workplace adaptation: a Walker and Avant concept analysis
Source: Front Public Health. 2025 Dec 10;13:1713343. doi: 10.3389/fpubh.2025.1713343 (PMC12727437; doi:10.3389/fpubh.2025.1713343)
Supplement: Supplementary file 1 [file Table_1.docx]

**Supplementary Table S1.** Detailed information on the included literature

| Author | Year | Title | Definition | Antecedent | Consequence |
| --- | --- | --- | --- | --- | --- |
| Zhang et al. | 2025 | The mediating effect of role clarity on the relationship between reality shock and workplace adaptability of newly graduated nurses | An individual’s self-regulation ability when solving unfamiliar, complex, and ambiguous problems can help individuals cope with pressure sources related to current and future career changes, which is of significant importance for personal career and social development. | Not applicable | Promotion of Personal Career Development |
| Xu et al. | 2025 | Analysis on the current situation and influencing factors of clinical leadership of new nurses with master’s degree | Not applicable | Not applicable | Optimization of Nursing Service Quality, Stabilization of Team Organizational Structure |
| Han et al. | 2024 | The real experience of workplace adjustment at different stages of the transition period for new nurses | An individual’s psychological state of establishing harmonious relationships to adapt to the work environment promotes the work behavior of new nurses. | Individual Intrinsic Adaptation Capital: professional competence | Promotion of Personal Career Development, Optimization of Nursing Service Quality, Stabilization of Team Organizational Structure |
| Yang et al. | 2024 | Turnover intention of newly graduated master degree nurses: a longitudinal study and multifactorial path analysis | A nurse’s ability to adapt to relevant work environments, regulations, and tasks, and establish harmonious relationships. | Organizational Socialization Strategies: workload management | Promotion of Personal Career Development |
| Sun et al. | 2024 | The Mediating Effect of Workplace Adaptation on Standardized Training of Nurses' Stress Quotient and Active Professional Behavior | Caregivers possess professional qualities, the ability to competently perform nursing duties, and the capacity to adapt to relevant work environments and establish harmonious relationships. | Individual Intrinsic Adaptation Capital: psychological capital | Promotion of Personal Career Development |
| Xue et al. | 2024 | Relationships Among Growth Mindset, Turnover Tendency, Workplace Adaptability and Essentials of Magnetism of New Nurses: A Moderated Mediation Model | An individual’s ability to follow the rules and regulations of the work environment and establish harmonious working relationships which can reflect the degree of match between individual characteristics and occupational requirements | Individual Intrinsic Adaptation Capital: psychological capital;  Organizational Socialization Strategies: work atmosphere | Promotion of Personal Career Development, Optimization of Nursing Service Quality, Stabilization of Team Organizational Structure |
| Baharum et al. | 2024 | The influencing factors of newly employed nurses' adaptation in Malaysia: a structural equation modelling assessment | Not applicable | Organizational Socialization Strategies: mentorship, work atmosphere;  Individual Intrinsic Adaptation Capital: psychological capital | Promotion of Personal Career Development |
| Liu et al. | 2023 | Study on the Correlation Between Psychological Capital and Workplace Adaptation of Standardized Training Nurses in Tertiary Hospital | The ability of caregivers to adapt to the work environment and establish harmonious relationships. | Sociodemographic factors:gender, educational background;  Individual Intrinsic Adaptation Capital: psychological capital | Promotion of Personal Career Development, Optimization of Nursing Service Quality |
| Kong et al. | 2023 | Research progress on adaptability of newly recruited nurses in the workplace | The ability of caregivers to adapt to the work environment and establish harmonious relationships, influencing nurses' work motivation and professional experience. | Sociodemographic factors: gender, educational background, upbringing;  Individual Intrinsic Adaptation Capital: psychological capital;  Organizational Socialization Strategies: work atmosphere, workload management | Promotion of Personal Career Development |
| Ukawa et al. | 2023 | Influence of Proactive Behaviors in Organizational Socialization of New Graduate Nurses on Their State of Workplace Adaptation | Actions taken by individuals to transform themselves or influence the environment to achieve a new state of equilibrium after experiencing a disruption of internal and external balance due to joining a new workplace, and the resulting harmonious equilibrium between the individual and the workplace. | Not applicable | Promotion of Personal Career Development |
| Zhao et al. | 2023 | Burnout among Junior Nurses: The Roles of Demographic and Workplace Relationship Factors, Psychological Flexibility, and Perceived Stress | Not applicable | Not applicable | Promotion of Personal Career Development |
| Baharum et al. | 2023 | Success factors in adaptation of newly graduated nurses: a scoping review | Not applicable | Organizational Socialization Strategies: workload management | Promotion of Personal Career Development |
| Liang et al. | 2022 | Status Quo of Medical Narrative Competence of New Nurses and Its Influencing Factors: A 422-case Study | An individual’s ability to adapt to relevant environmental regulations and establish harmonious relationships. | Not applicable | Promotion of Personal Career Development |
| Wang et al. | 2022 | Transition shock and workplace adaptability in newly graduated nurses: multiple mediating roles of resilience and person-organization fit | The ability to adapt to relevant environmental regulations at work and establish harmonious relationships. | Individual Intrinsic Adaptation Capital: psychological capital | Promotion of Personal Career Development, Optimization of Nursing Service Quality, Stabilization of Team Organizational Structure |
| Ji et al. | 2022 | Workplace adaptability and its influencing factors among newly graduated nurses | Adaptation to the work environment. | Sociodemographic Factors: gender;  Organizational socialization strategies: humanistic care, work atmosphere, workload management | Promotion of Personal Career Development, Optimization of Nursing Service Quality, Stabilization of Team Organizational Structure |
| Wu et al. | 2022 | Moderating effect of proactive personality on workplace adaptation and nursing behavior of standardized training nurses | The ability of caregivers to adapt to the work environment and establish harmonious relationships. | Organizational Socialization Strategies: work atmosphere | Promotion of Personal Career Development, Optimization of Nursing Service Quality |
| Kitajima et al. | 2022 | Workplace Adaptation Behavior and State Scales for New Nurses: Testing for Reliability and Validity | This refers to actions an individual takes to transform themselves or influence the environment to achieve a new state of equilibrium after experiencing a disruption of internal and external balance upon joining a new workplace, and the resulting state of harmonious equilibrium between the individual and the workplace. | Individual Intrinsic Adaptation Capital: professional competence, interpersonal skills, psychological capital;  Organizational Socialization Strategies: mentorship | Promotion of Personal Career Development |
| Kawakami et al. | 2022 | Difference between perceptions of preceptors and newly graduated nurses regarding delay in professional growth: a thematic analysis | Not applicable | Individual Intrinsic Adaptation Capital: professional competence, interpersonal skills | Promotion of Personal Career Development |
| Ge et al. | 2021 | Current Status of Workplace Adjustment of New Nurses with Bachelor Degree or above and Its Influence Factors: A 471-case Study | The ability to adapt to relevant environmental regulations at work and establish harmonious relationships. | Sociodemographic Factors: upbringing, political affiliation；  Organizational Socialization Strategies: humanistic care | Optimization of Nursing Service Quality |
| Liu et al. | 2021 | Chinese version of Nurses' Workplace Adaptability Scale and its reliability and validity test | The ability to adapt to relevant environmental regulations at work and establish harmonious relationships. | Individual Intrinsic Adaptation Capital: psychological capital | Promotion of Personal Career Development, Optimization of Nursing Service Quality, Stabilization of Team Organizational Structure |
| Kitajima et al. | 2019 | Surface and Content Validity of Workplace Adaptive Behavior Scale and Workplace Adaptive State Scale for New Nurses | Actions taken by individuals to transform themselves or influence the environment to achieve a new state of equilibrium after experiencing a disruption of internal and external balance due to joining a new workplace, and the resulting harmonious equilibrium between the individual and the workplace. | Individual Intrinsic Adaptation Capital: interpersonal skills, psychological capital | Promotion of Personal Career Development |
| Dames et al. | 2019 | THRIVEable work environments: A study of interplaying factors that enable novice nurses to thrive | Not applicable | Organizational Socialization Strategies: workload management | Promotion of Personal Career Development |
| Kitajima et al. | 2018 | Qualitative and Inductive Research on the Workplace Adaptation of New Nurses | An individual’s action of changing him/herself and influencing the environment after experiencing the collapse of internal and external balance upon joining a new workplace to achieve a new equilibrium as well as the resulting stable equilibrium between the individual and the workplace | Individual Intrinsic Adaptation Capital: professional competence, interpersonal skills, psychological capital;  Organizational Socialization Strategies: mentorship | Promotion of Personal Career Development |
| Nagata et al. | 2017 | Factors Related to Workplace-adaptation of Rookie Male Nurses | Having successfully navigated the socialization phase up to six months after employment and reached the second year. | Individual Intrinsic Adaptation Capital: professional competence, interpersonal skills | Not applicable |
| Kuriki et al. | 2016 | Related a workplace adaptation and burn-out of new graduate nurses who is experienced in working different type of industry | The establishment of an identity as a nurse by new graduate nurses internalizing their roles, behaviors, and values as professionals, and integrating themselves with the professional group. | Not applicable | Promotion of Personal Career Development |
| Ishii et al. | 2015 | Guidance Methods for Job Adaptation of Newly Graduated Nurses –Differences between the Recognition of Newly Graduated Nurses Unable to Adapt to the new Job, Newly Graduated Nurses Who Could, and Preceptors– | New graduate nurses adapting to workplace relationships with superiors, colleagues, patients, workplace autonomy, work environment, job relationships, and workplace regulations. | Organizational Socialization Strategies: mentorship | Promotion of Personal Career Development |
| Fujimoto et al. | 2013 | Re-examination of nurses' workplace adaptability scale | Within the workplace where nurses are employed, an individual becoming accustomed to the norms of the workplace environment and achieving harmonious relationships. | Sociodemographic Factors: gender;  Individual Intrinsic Adaptation Capital: interpersonal skills | Not applicable |
| Asako | 2012 | A Study on Job-adjustment for New Graduate Nurses in Acute Hospitals —With Special Reference to Factors that Hamper Work Continuation— | Used to describe a state of performing duties without excessive tension and adapting to senior nurses and the work environment. | Not applicable | Promotion of Personal Career Development |
| Sunami et al. | 2012 | Factors affecting Work Adjustment Process of Newly Graduate Nurses: A Literature Review | The process by which new nurses become familiar with their affiliated workplace, learning and acquiring the roles and attitudes required by that workplace. | Individual Intrinsic Adaptation Capital:professional competence, interpersonal skills, and psychological capital;  Organizational Socialization Strategies: mentorship | Promotion of Personal Career Development |
| Onoda et al. | 2012 | Longitudinal Study on Newly Graduated Nurses’ Adjustment to the Workplace and Factors that Exert an Influence | Maintaining a good physical and mental state while adapting to work and the work environment. | Individual Intrinsic Adaptation Capital:professional competence | Promotion of Personal Career Development |
| Miwa et al. | 2010 | Analysis of factors relating to adaptability of newly graduated nurses in work places | Within the interaction with the environment from approximately 3 to 6 months after employment, new graduate nurses internalize role behaviors and values as nursing professionals, integrating themselves with the professional group and themselves to establish an identity as a nurse. | Individual Intrinsic Adaptation Capital:professional competence;  Organizational Socialization Strategies: mentorship, work atmosphere | Not applicable |
| Hirata et al. | 2008 | New Nurses’ Adjustment to Initial Employment – The Difficult Experience of Working the Night Shift– | Adaptation as a human being to their profession, adaptation to lifestyle with shift work, and adaptation in terms of skills to workplace duties. | Individual Intrinsic Adaptation Capital: psychological capital;  Organizational Socialization Strategies: workload management | Promotion of Personal Career Development |
| Furuichi et al. | 2006 | Job Adjustment among New Graduate Nurses | Developing ways of thinking and behaving that are suited to the workplace environment. | Individual Intrinsic Adaptation Capital: professional competence | Promotion of Personal Career Development |
| Paker et al. | 2003 | Adequacy of support for new graduates during their transition into the workplace: a Queensland, Australia study | Not applicable | Organizational Socialization Strategies | Not applicable |
